# The RpTOE1-*RpFT* Module Is Involved in Rejuvenation during Root-Based Vegetative Propagation in *Robinia pseudoacacia*

**DOI:** 10.3390/ijms23095079

**Published:** 2022-05-03

**Authors:** Zijie Zhang, Jie Liu, Sen Cao, Qi Guo, Yuhan Sun, Dongsheng Niu, Cui Long, Yingming Fan, Yun Li

**Affiliations:** 1 Engineering Technology Research Center of Black Locust of National Forestry and Grassland Administration, National Engineering Laboratory for Tree Breeding, Key Laboratory of Genetics and Breeding in Forest Trees and Ornamental Plants of Ministry of Education, College of Biological Sciences and Technology, Beijing Forestry University, Beijing 100083, China; zijiezhang@bjfu.edu.cn (Z.Z.); liujie3190266@bjfu.edu.cn (J.L.); caosen0419@bjfu.edu.cn (S.C.); guoqi@bjfu.edu.cn (Q.G.); sunyuhan@bjfu.edu.cn (Y.S.); longcui@bjfu.edu.cn (C.L.); ymfan@bjfu.edu.cn (Y.F.); 2 Black Locust Seed Orchard of Jixian County, Linfen 042200, China; niudongsheng@163.com

**Keywords:** vegetative propagation, rejuvenation, *Robinia pseudoacacia*, AP2-like TF

## Abstract

Vegetative propagation is an important method of reproduction and rejuvenation in horticulture and forestry plants with a long lifespan. Although substantial juvenile clones have been obtained through the vegetative propagation of ornamental plants, the molecular factors that regulate rejuvenation during vegetative propagation are largely unknown. Here, root sprouting and root cutting of *Robinia pseudoacacia* were used as two vegetative propagation methods. From two consecutive years of transcriptome data from rejuvenated seedlings and mature trees, one gene module and one miRNA module were found to be specifically associated with rejuvenation during vegetative propagation through weighted gene co-expression network analysis (WGCNA). In the gene module, a transcription factor-encoding gene showed high expression during vegetative propagation, and it was subsequently named *RpTOE1* through homology analysis. Heterologous overexpression of *RpTOE1* in wild-type *Arabidopsis* and *toe1* *toe2* double mutants prolonged the juvenile phase. The qRT-PCR results predicted *RpFT* to be a downstream gene that was regulated by RpTOE1. Further investigation of the protein-DNA interactions using yeast one-hybrid, electrophoretic mobility shift, and dual luciferase reporter assays confirmed that RpTOE1 negatively regulated *RpFT* by binding directly to the TOE binding site (TBS)-like motif on its promoter. On the basis of these results, we showed that the high expression of *RpTOE1* during vegetative propagation and its inhibition of *RpFT* played a key role in the phase reversal of *R. pseudoacacia*.

## 1. Introduction

Vegetative propagation is a form of asexual reproduction in which new plants form from the vegetative parts of the parent plant [1]. Unlike sexual propagation, which uses seeds to obtain new plants from zygotic embryogenesis, vegetative propagation uses meristematic, undifferentiated cells to form juvenile (juvenile-like) replicas from the ortet [2] (mother plant used as the source of tissue for regeneration). For ornamental plants, mass genetic copies can be obtained more quickly and easily by vegetative propagation. Cuttings are the most widespread and popular method of vegetative propagation, due to their simplicity [3]. However, some cuttings root easily, while some are recalcitrant, even with the use of growth regulators [4]; physiological processes resulting from age, ontogenetic phase, condition of the stock, and genetic background can greatly affect rooting [5,6,7]. The decreased rooting ability of cuttings caused by maturation has been an essential issue for the efficiency of asexual reproduction.

The adult phase in plants can be reversed under certain conditions (defined as rejuvenation), in which the shoot meristem regains some or all of its juvenile characteristics after reproductive maturity is attained [8,9]. Plant rejuvenation can be achieved by applying phytohormones, repeated grafting, micropropagation, cutting, and others [10,11]. Restoring the decreased rooting ability of mature plants through rejuvenation has become a key method for the application of asexual propagation in plants [12]. *Cichorium intybus* and *Lactuca sativa* plants re-established a juvenile phase by a further cycle of regeneration in vitro. The survival rate, flowering frequency, and seed yield of rejuvenated plants were improved [13]. In trees, it was easier for juvenile explants to root, and reversing maturation through vegetative propagation was usually necessary to restore rooting capacity [14]. The phenomenon of juvenility in vegetative propagation is attracting widespread attention. The switches in cell fate during vegetative propagation, accompanied by the transfer of gene expression from somatic cells to new developmental pathways, were characterized by remarkable changes in specific gene expression [15]. Therefore, exploring how the ortet resets its gene expression patterns is essential for understanding the rejuvenation of vegetative propagation.

A previous study found that the genetic regulation mechanisms involved in the juvenile and rejuvenation stages were similar, and both stages were mainly regulated by small RNAs [16]. More recent works in plants have shown that miR156 and miR172, as well as their respective *SQUAMOSA PROMOTER BINDING PROTEIN-LIKE* (*SPL*) and *APETALA2* (*AP2*) transcription factor targets, play a central role in this process [17,18,19]. MiR156 was highly expressed in the juvenile phase, and this expression decreased with the increasing plant. The inhibition of its targets gradually weakened and then determined the juvenile-to-adult transition [20]. Overexpression of miR156 prolonged the juvenile stage of plants, while inhibition of miR156 accelerated maturation [21]. In *Arabidopsis*, miR156 further regulated *FLOWERING LOCUS T* (*FT*) expression by inhibiting *SPL3* to control temperature-responsive flowering [22]. Other regulators, such as miR172 and its targets, are also involved in plant developmental transitions [23,24,25]. MiR156 cooperates with miR172 to orchestrate diverse age-related developmental events. MiR172 acts downstream of miR156 and shows a temporal expression pattern that is the opposite to that of miR156. In *Arabidopsis*, miR156 inhibited the transcription of miR172 by SPL9/10 and then repressed the expression of *AP2*-like genes [18,21].

The AP2-LIKE transcription factor (TF) family consists of six members, APETALA2 (AP2), TARGET OF EAT1 (TOE1), TOE2, TOE3, SCHNARCHZAPFEN (SNZ), and SCHLAFMUTZE (SMZ), in *Arabidopsis* [26]. All six AP2-like TFs act as repressors of flowering [27]. Overexpression of miR172 led to an early flowering phenotype, while increasing its targets, *SMZ or SNZ*, led to an opposite phenotype [28]. The gain-of-function or loss-of-function phenotype of single gene mutations revealed a high degree of functional redundancy within these families [29]. TOEs can delay flowering as repressors of variable flowering enhancers or integrators [30,31,32]. The *toe1* mutant flowered significantly earlier than the wild type, and the phenotype was further enhanced in *toe1 toe2* double mutants under long-day conditions [33]. Overexpression of AP2, SMZ, SNZ, TOE1 or TOE2 resulted in late flowering [26,34], whereas the flowering of *smz snz toe1 toe2* mutants occurred earlier than that of any single or double mutant [27,35]. The same results were also found in the regulation of trichome initiation in *Arabidopsis* [29]. Most studies on AP2-LIKE TFs in annual plants focus on ontogenetic aging, and their role in the developmental phase reversal of woody plants has not been revealed.

The florigen-encoding gene *FT* determines the flowering time of model plant. It is transcribed in the vasculature of leaves, and its protein is transported to the shoot apical meristem (SAM) to induce cell differentiation [36]. Further studies showed that TOE1 and SMZ were able to bind directly to the promoter of *FT* to prevent flowering. The protein binding assay confirmed that the putative TOE binding site (TBS) was AACCTACGA, and TOE1 can also recognize DNA sequences of TBS-like and AT-rich elements [30]. The linear miR156-SPL9-miR172-TOE1/TOE2 is a cascaded, cooperative transcriptional regulator of flowering time [37]. The study also showed that *TOE1* and *TOE2* positively regulated miR172 by a negative feedback loop [18]. The interaction of miR156-SPLs-miR172 was conserved across spermatophytes [38,39]. Researchers found similar expression patterns of miR156 and miR172 and their target genes in several woody plants [40]. In *Jatropha curcas*, overexpression of miR172 resulted in early flowering, abnormal flowers, and altered leaf morphology [41]. A study in poplar confirmed that the transition from the vegetative to reproductive stage was regulated by the miR156-SPL pathway [42]. Studies of phase change have provided clues for the study of phase reversal regulation in vegetative propagation. Rejuvenation is a reverse process of maturation, but it has not been revealed which nodes that regulate plant maturation are reversed during the rejuvenation event of woody plants under asexual reproduction. In strawberry tissue culture, the results suggested that high expression of miR156 was the main reason for the rejuvenation of micropropagation [43]. However, by inducing miR156 expression in the adult leaves of *A. thaliana*, the regenerated plants could reconstruct only some juvenile traits, but could not reverse chronological age [44]. A recent study showed that the rate of decline in miR156 was correlated only with developmental age rather than with chronological age [45]. Therefore, in addition to miR156, other unknown regulatory factors may be involved in the rejuvenation of plant vegetative propagation.

Compared with annual plants, the life cycle of perennial plants is longer, and vegetative propagation is the main reproductive mode of some woody plants. Vegetatively propagated plants showed a degree of juvenility compared with the ortet [46]. However, under the same genetic background, the internal mechanism of rejuvenation during vegetative propagation is still unclear. A study on woody plants found that the closer the explants were to the root, the better the rooting effect was [47]. In *E. camaldulensis*, adventitious rooting was significantly higher in coppice shoot cuttings than in stem cuttings [48]. Choosing roots as explants for vegetative propagation has become the ideal choice for difficult rooting species. *Robinia pseudoacacia* is a versatile landscape tree and is increasingly grown as a horticultural species because of its ornamental attractiveness. In this study, two types of root-propagated seedlings of the same mature *R. pseudoacacia* trees were used as rejuvenated plants to explore the specific mechanisms of rejuvenation during the vegetative propagation of woody plants. Seedlings developed from seeds of the same mature trees were used as controls for sexual propagation to exclude the juvenile factors caused by sexual reproduction. Comparative analysis of the transcriptome between rejuvenated seedlings and mature trees was performed for two consecutive years. Co-expression network analysis identified a gene that plays a critical role in the juvenility maintenance of root-based vegetative propagation; this gene was named *RpTOE1.* Heterologous overexpression of *RpTOE1* in wild-type (WT) *A. thaliana* and *toe1 toe2* double mutants prolonged the juvenile phase. Using protein-DNA assays, we further found that RpTOE1 negatively regulated *RpFT* by directly binding to the TBS-like motif on its promoter. In our study, RpTOE1, a key factor in the developmental phase reversal between rejuvenated seedlings and mature trees in *R. pseudoacacia,* was mined and experimentally verified to interact with *RpFT* to participate in rejuvenation during vegetative propagation. Its high expression in young tissues and plants and low expression in mature tissues and plants provide novel insights into the evaluation of material maturity during the vegetative propagation of woody plants.

## 2. Results

### 2.1. Experimental Design for Exploring the Genetic Regulation of Rejuvenation during Vegetative Propagation

**Figure 1:**
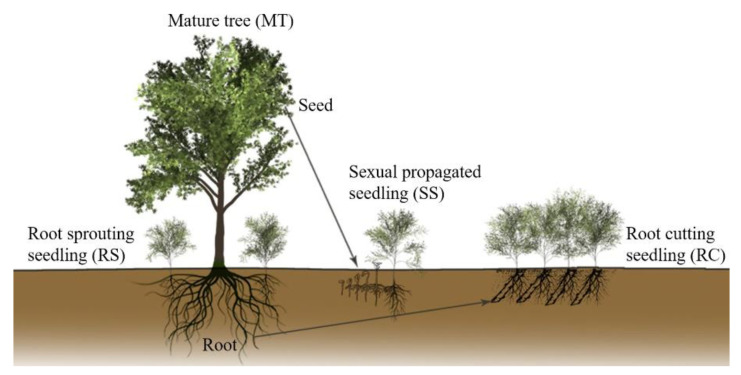
Schematic drawing of a mature tree and its seedlings. Root sprouting seedlings (RSs) and root cutting seedlings (RCs) were propagated asexually from the roots of mature trees (MTs). Sexually propagated seedlings (SSs) developed from half-sibling seeds of MTs.

Mature trees (MTs) were identified in the *R. pseudoacacia* plantation. For each tree, age was determined by coring at breast height. Three trees of the same age were chosen for subsequent experiments and analysis. The root sprouting seedlings (RSs) were obtained by scraping the roots of a MT in situ, the root cutting seedlings (RCs) were obtained by cutting the roots of the same MT in vitro, and the seeds of the same MT were sown to obtain sexually propagated seedling (SS) populations as the control for sexual propagation (Figure 1). One-year-old RS, RC, and SS plants, and the MT that produced them were named FRS, FRC, FSS, and FMT, respectively. Two-year-old seedlings and MTs were named SRS, SRC, SSS, and SMT. Leaf samples were collected for transcriptome analysis.

### 2.2. Comparative Transcriptome Profiles between MTs and Seedlings over Two Years

**Figure 2:**
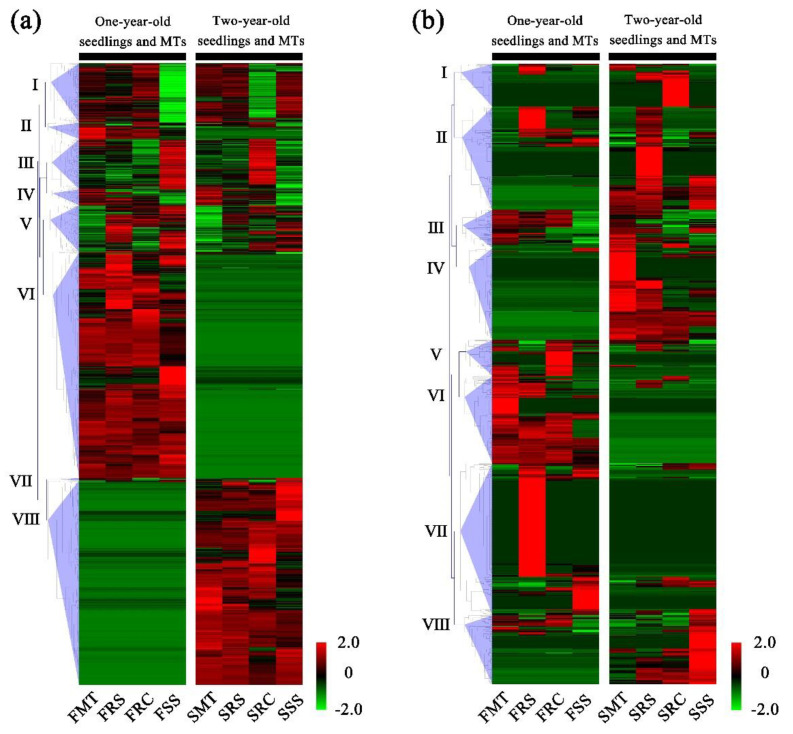
Heatmaps of differentially expressed genes and differentially expressed miRNAs. (**a**) Heatmap of differentially expressed genes between mature trees and one- and two-year-old seedlings; (**b**) Heatmap of differentially expressed miRNAs between mature trees and one- and two-year-old seedlings. FMT, mature trees in the first year of sampling; FRS, one-year-old root sprouting seedlings; FRC, one-year-old root cutting seedlings; FSS, one-year-old seed-derived seedlings; SMT, mature trees in the second year of sampling; SRS, two-year-old root sprouting seedlings; SRC, two-year-old root cutting seedlings; and SSS, two-year-old seed-derived seedlings. Each group contained three biological replicates (three different trees).

We identified a total of 4495 differentially expressed genes (DEGs) and 735 differentially expressed miRNAs (DEMs) between one-year-old seedlings and FMT, and 4145 DEGs and 748 DEMs between two-year-old seedlings and SMT (Figure 2, Tables S1 and S2). These DEGs and DEMs were divided into several clusters, and comparisons between the mature trees and seedlings revealed specific expression profiles. A total of 2420 (36.34%) and 2187 (32.84%) genes (mostly in Clusters Ⅵ and Ⅷ) were only significantly differentially expressed in the one-year-old and two-year-old seedlings, respectively (Figure 2a). Interestingly, in the expression profile of DEMs, many miRNAs only showed differences in specific plants (Figure 2b). For example, most of the miRNAs in Cluster Ⅰ were highly expressed in SRC, and most of the miRNAs in Cluster Ⅱ were highly expressed in FRS and SRS. The miRNAs in Cluster Ⅶ also showed the same expression pattern in FRS and FSS (Figure 2b). This suggested that some of the miRNAs may be specifically associated with reproductive methods. These expression profiles revealed the expression patterns of genes and miRNAs in plants of specific age and those generated by different reproductive methods. To verify the RNA-seq data, the expression of 10 genes and 10 miRNAs was validated by qRT-PCR and stem-loop qRT-PCR using SMT, SRC, and SSS samples that were the same as those used for RNA-seq (Figure S1). Except for one miRNA in the SSS sample and one gene and one miRNA in the SRC sample that showed opposite trends, the results were generally consistent with the sequencing data.

### 2.3. Identification of Gene and miRNA Co-Expression Modules Related to Vegetative Propagation in R. pseudoacacia

**Figure 3:**
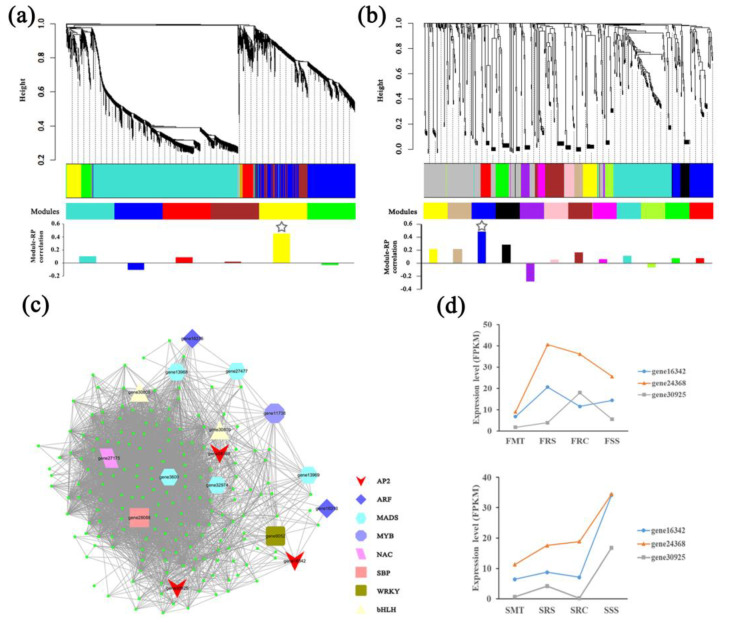
Weighted gene co-expression network analysis (WGCNA) and expression patterns of differentially expressed genes and miRNAs. (**a**) WGCNA was conducted on a total of 6659 differentially expressed genes (*p* < 0.05) between the mature trees and at least one type of seedling over two years. Modules of co-expressed genes and their correlation with root-based vegetative propagation (RP) were assigned color bars beneath each dendrogram; (**b**) WGCNA was conducted on a total of 1169 differentially expressed miRNAs (*p* < 0.05) between the mature trees and at least one type of seedling over two years. Modules of co-expressed miRNAs and their correlation with root-based vegetative propagation (RP) were assigned color bars beneath each dendrogram; (**c**) The gene regulatory network of the Gyellow module. The large polygons indicate significantly differentially expressed TFs, while other genes are shown with small green circles; (**d**) Expression levels of three AP2-like TFs in one- and two-year-old seedlings and mature trees. Three biological replicates were sequenced for each sample group. The stars in (**a**,**b**) show the modules with the highest correlation coefficients. FMT, mature trees in the first year of sampling; FRS, one-year-old root sprouting seedlings; FRC, one-year-old root cutting seedlings; FSS, one-year-old seed-derived seedlings; SMT, mature trees in the second year of sampling; SRS, two-year-old root sprouting seedlings; SRC, two-year-old root cutting seedlings; and SSS, two-year-old seed-derived seedlings.

To obtain an overall understanding of the complex datasets, we applied weighted gene co-expression network analysis (WGCNA) to identify the hub gene and miRNA regulatory networks under vegetative propagation. A total of 6659 genes and 1169 miRNAs that were differentially expressed (*p* < 0.05) between the mature trees and at least one type of seedling over two years were analyzed, based on the expression profiles of all 24 samples. Genes and miRNAs were clustered by expression patterns, as indicated by the dendrogram, and modules are indicated in different colors (Figure 3a,b). We found that 6 and 12 modules contained 3710 (55.71%) genes and 418 (35.76%) miRNAs, respectively.

Furthermore, the modules were correlated with asexual and sexual reproduction methods to identify the modules specifically related to rejuvenation during vegetative propagation. Among these, the Gyellow module contained 201 genes and the Mblue module contained 63 miRNAs that were found to be most highly correlated with asexual propagation but not with sexual propagation (Figure S2). These results suggested that the Gyellow and Mblue modules contained the main genes and miRNAs sensitive to root-based vegetative propagation in all seedlings. The Gyellow module contained 16 predicted TF-coding genes, three of which were age-sensitive AP2 TFs (Figure 3c). Moreover, these three *AP2*-like genes were highly expressed in juvenile seedlings (Figure 3d), among which gene24368 had the highest expression and was selected as a hub gene for further functional validation. The Mblue module of the miRNA network contained 24 known miRNAs and 39 novel miRNAs (Table S3). Among the 24 known miRNAs, miR156/157 and miR172, which have been shown to regulate phase transition in model plants, were not found. The miRNAs in Mblue and the genes in Gyellow were also used for target prediction, and a total of 37 pairs of predicted targeting relationships were obtained (Figure S3).

### 2.4. Identification and Characterization of RpTOE1

The full-length sequence of gene24368 was cloned from the leaves of *R. pseudoacacia*. Based on sequencing, the gene was predicted to be 1362 bp long, and it encoded a 49.94-kDa protein containing 453 amino acids (aa). Homology analysis revealed a high degree of identity with the AtTOE1 protein. A phylogenetic tree constructed by gene24368 and the *Arabidopsis* AP2 subfamily also showed that gene24368 belonged to the AP2 family and showed the highest similarity with AtTOE1 (Figure S4); therefore, we named this gene *RpTOE1*. Then, we compared the RpTOE1 protein with *Arabidopsis* TOE proteins, and two AP2 domains were found; these domains were located at residues 133–195 and 225–273. A nuclear localization signal (NLS) sequence was also contained in the RpTOE1 protein and was located at residues 121–129 (Figure S5).

### 2.5. Heterologous Overexpression of RpTOE1 Prolongs the Vegetative Stage in Arabidopsis

**Figure 4:**
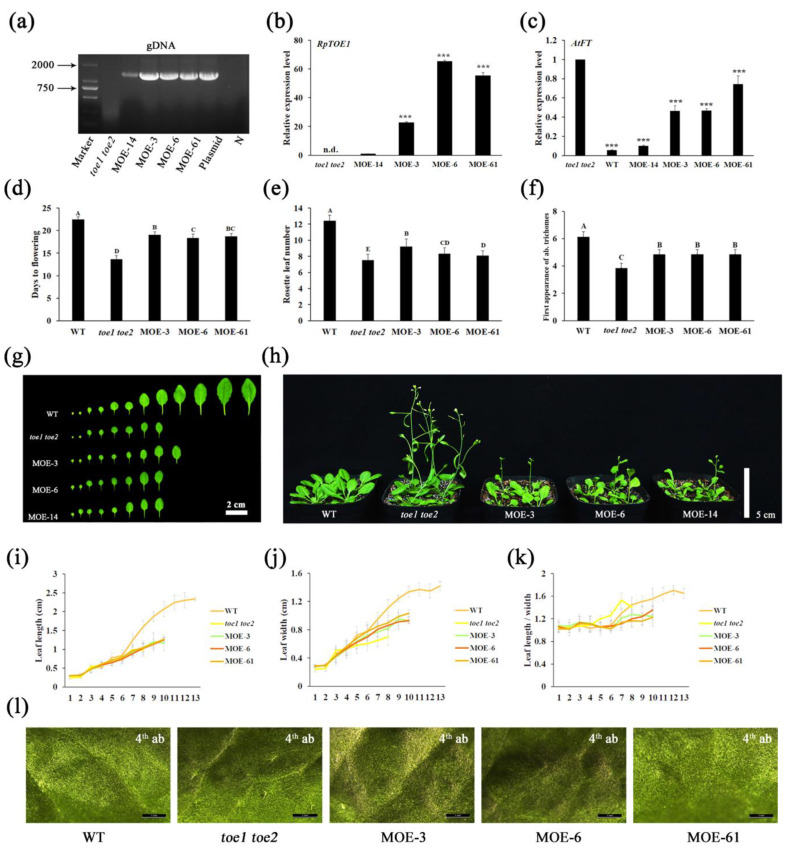
Overexpression and identification of *RpTOE1* in *Arabidopsis toe1 toe2* mutants. (**a**) PCR analysis of *RpTOE1* genes from the genomic DNA of *toe1 toe2* double mutants and four transgenic lines; (**b**) Relative expression of *RpTOE1* in *toe1 toe2* double mutants and four transgenic lines with *AtACTB* as an internal control using qRT-PCR. Data represent the average of three biological replicates; (**c**) Relative expression of the *AtFT* gene in *toe1 toe2* double mutants, wild-type (WT), and four transgenic lines. (**d**) Days to flowering of WT, *toe1 toe2* double mutants and transgenic lines; (**e**) Rosette numbers when floral primordia were visible; (**f**) The abaxial (ab) trichome statistics. The first leaf with abaxial trichomes was scored (*n*>20); (**g**) All rosette leaves on one plant of WT, *toe1 toe2* double mutants and transgenic lines when floral primordia were visible in *toe1 toe2* double mutants; (**h**) Phenotype of the WT, *toe1 toe2* double mutants and *RpTOE1*-overexpressing plants in *toe1 toe2* double mutants; (**i**–**k**) Leaf shape measurement when floral primordia were visible. The x-axes indicate the order of leaves. The y-axes indicate leaf length (**i**); leaf width (**j**); and leaf length/width ratio (**k**); (**l**) The abaxial trichome phenotype. MOE-14, MOE-3, MOE-6, and MOE-61represent *RpTOE1*-overexpressing plants in *toe1 toe2* double mutants. N, negative control using water as a template. The expression levels were quantified in 15-day-old seedlings. *** significant differences between the MOE-14 line and *toe1 toe2* double mutants based on Student’s *t*-test (*p* < 0.001). n.d. = not detected. Capital letters designate groups that significantly differ (*p* < 0.01). Error bars represent SEM. Bar = 2 cm in (**g**), bar = 5 cm in (**h**)**,** and bar = 1 mm in (**l**). *n* > 30 in (**d**, **e**, **i**–**k**).

To elucidate how *RpTOE1* functions in rejuvenation, *RpTOE1* was transferred into WT *Arabidopsis* and *toe1 toe2* double mutants by the floral dip method. After single-copy transformation screening, PCR and qRT-PCR analysis, four independent transgenic overexpression lines in WT (OE) and the mutants (MOE) were obtained separately (Figure 4a,b and Figure S6a,b). Three independent homozygous lines with high *RpTOE1* expression in the T_3_ generation were used for phenotypic assays. Compared to WT, the 35S::*RpTOE1* transgenic plants exhibited no visible alteration in flowering time, serration on the leaf margin, rosette leaf number, or leaf shape (Figure S6c–h,j), but exhibited a late abaxial trichome phenotype (Figure S6i,k). Although overexpression of *RpTOE1* in WT only changed one aspect of the juvenile traits, it also suggested that *RpTOE1* could prolong the juvenile phase to a certain extent.

The *toe1 toe2* double mutants show an obvious early-flowering phenotype, and the early-flowering phenotype can be partially restored with overexpression of *RpTOE1* (Figure 4d,h). Compared to the double mutants, the days to flowering and rosette number of the 35S::*RpTOE1* transgenic plants in mutant backgrounds were higher than the mutants alone but lower than WT (Figure 4d,e). The abaxial trichome phenotype of the transgenic plants was also intermediate between the mutants and WT (Figure 4f,l). The mutants and transgenic plants were similar in serrations on the leaf margin, leaf length, leaf width, and leaf length/width, and these values were all lower than those for WT (Figure 4g,i–k). To further explore the function of RpTOE1, we examined the expression of *AtFT* in WT, *toe1 toe2* double mutants and transgenic plants. Interestingly, consistent with the phenotypes for days to flowering, rosette number, and abaxial trichome, the expression of *AtFT* also showed that the transgenic plants were intermediate between the WT and mutants (Figure 4c).

### 2.6. RpTOE1 Can Directly Bind to the Promoter of RpFT and Inhibit Its Expression

To further explore the regulatory process involving *RpTOE1*, qRT-PCR assays were performed in black locust, *Arabidopsis toe1 toe2* double mutants, and transgenic plants. The expression levels of *RpTOE1* in different tissues of SRC, SSS, and SMT were detected. The results showed that the expression level of *RpTOE1* was the highest in the roots of the juvenile and adult plants (Figure S7). In two-year-old seedlings, the expression level of *RpTOE1* was lower in leaves than in roots, followed by stems, and was the lowest in shoot tips. However, there was no significant difference in the expression of *RpTOE1* in the shoot tips, stems, and leaves of mature trees. In *Arabidopsis*, compared to that in the *toe1 toe2* double mutants, the expression level of *AtFT* was decreased in phenotypic rescue lines (Figure 4c). Combined with the results of previous studies in *A. thaliana*, *RpTOE1* likely prolongs the vegetative stage of mutants by regulating the expression of the *FT* gene.

To determine whether RpTOE1 directly downregulated the expression of *RpFT* in black locust, an ~2-kb promoter sequence of *RpFT* was cloned from black locust leaves (Dataset S1). Within the promoter region, we found a TBS-like motif located 1447 bp upstream of the translation start codon (Dataset S1). Furthermore, protein-DNA assays were used to verify the binding site of RpTOE1.

**Figure 5:**
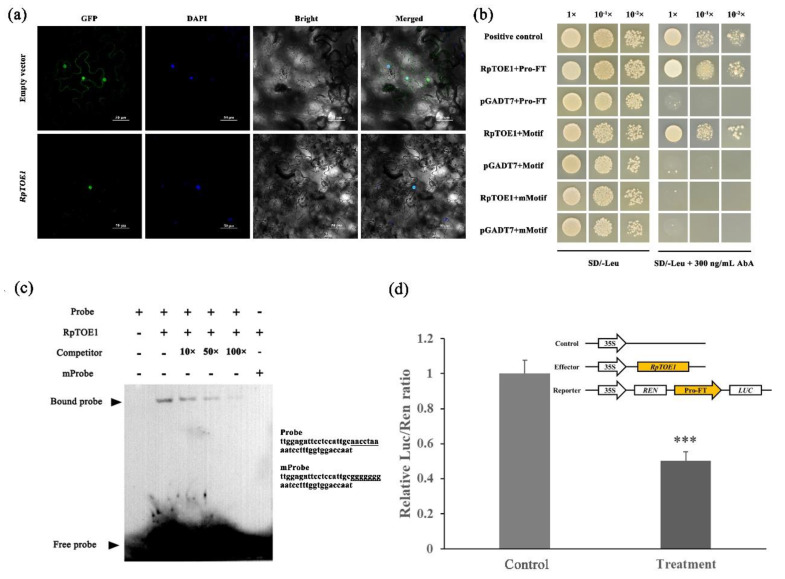
Transcription factor RpTOE1 directly repressed *RpFT* via a TBS-like motif. (**a**) Subcellular localization of RpTOE1, as determined by the expression of 35S:*RpTOE1*-GFP constructs in tobacco leaves. The empty vector was used as the control. DAPI is a marker for localization in the nucleus (*n* = 3). Bars, 50 μm; (**b**) Yeast one-hybrid assays. Pro-FT indicates the ~2-kb promoter of *RpFT*. Motif indicates that the 300-bp sequence includes the TBS-like motif in the *RpFT* promoter. mMotif indicates the mutated form of the TBS-like motif. pGADT7-p53 and p53-pAbAi were constructed as positive controls (*n* = 3); (**c**) Electrophoretic mobility shift assay. The probe sequence occurs in the *RpFT* promoter and harbors the TBS-like motif (Probe). The mutated probe (mProbe) was produced by replacing the TBS-like motif with ‘ggggggg’. ‘+’ and ‘-‘ indicate whether the reagents were present in the lane (*n* = 3); (**d**) Dual-luciferase assay. Data showing the Luc/Ren ratios for co-expression of the empty effector control (Control) and for co-expression of the effector and reporter (Treatment) (*n* = 6). Error bars represent SD. *** Significant differences from the control based on Student’s *t*-test (*p* < 0.001).

AP2 transcription factors are usually nuclear localization proteins. To explore the function and location of RpTOE1, the subcellular localization of GFP-labeled protein was observed in tobacco leaves. As shown in Figure 5a, labeled RpTOE1 protein was observed in the nucleus, while the control was observed both in the nucleus and around the cytoplasm. The ~2-kb promoter sequence of *RpFT* (named Pro-FT), a 300-bp sequence containing a TBS-like motif in Pro-FT (named Motif), and the 7-bp TBS-like motif mutated to ‘ggggggg’ (named mMotif) were used for yeast one-hybrid (Y1H) assays (Dataset S1). We found that RpTOE1 could interact with the Pro-RpFT and TBS-like motifs in yeast, while there was no interaction between RpTOE1 and the mutant motif (Figure 5b). The interaction between RpTOE1 and the TBS-like motif was also verified by electrophoretic mobility shift assays (EMSAs). The 45-bp sequence containing the TBS-like motif in the promoter of Pro-RpFT was designed as the probe (named Probe), while the TBS-like motif mutated to ‘ggggggg’ was designed as a mutated probe (named mProbe) (Figure 5c). The recombinant His-RpTOE1 fusion protein was purified after prokaryotic expression. The results showed that the recombinant His-RpTOE1 protein could bind to biotin-labeled probes containing the TBS-like motif, but not to the mutated TBS-like probe (Figure 5c). When an unlabeled *RpFT* promoter fragment was added as a cold competitor, the mobility shift was effectively abolished in a dose-dependent manner. These results clearly indicated that the binding of RpTOE1 to the *RpFT* promoter was specific.

Transient dual-luciferase assays (dLUC) were further used to determine the transactivation of *RpFT* by RpTOE1. *RpFT* promoters ~2-kb in length were fused with the LUC reporter. The pGreenⅡ 62-SK containing *RpTOE1* acted as an effector, and the empty vector was used as a control. The LUC/REN ratio was detected after the reporter and effector plasmids were co-expressed in tobacco leaves (Figure 5d). Compared with the control, the treatment significantly reduced the LUC/REN ratio. This result suggested that RpTOE1 could downregulate the expression of *RpFT* in vivo.

## 3. Discussion

Asexual propagation, as an important reproductive method in horticulture and forestry, has been widely used in genetic stability maintenance and factory production [49,50]. Phenotypic and physiological changes between perennial, vegetatively propagated plants, and ortets have revealed highly ordered rejuvenation events involving development, photosynthesis, and stress resistance [46,51]. Black locust is an excellent candidate for horticultural uses because it is drought tolerant, fast growing, and adaptable to many sites and climates [52]. In this study, to investigate the internal regulatory factors of rejuvenation during asexual reproduction, two types of asexually propagated seedlings were created, based on the roots of mature *R. pseudoacacia* trees (Figure 1). Comparative transcriptome data for two consecutive years demonstrated that many genes showed continuous variation between mature trees and rejuvenated seedlings. However, many miRNAs showed discontinuous variation, with only high (or low) expression in certain individuals (Figure 2b). This phenomenon may be associated with the different ways in which genes and miRNAs function during regulation, and some miRNAs may only play a regulatory role in specific reproductive patterns [53,54]. Based on the co-expression network analysis of transcriptome profiles for two consecutive years between rejuvenated seedlings and mature trees, we identified a gene module and a miRNA module highly specifically associated with rejuvenation during root-based asexual propagation (Figure 3a,b). In the gene module (Gyellow), we found some TFs closely related to plant development, including three AP2-like TFs (Figure 3c). In this study, we focused on the investigation of one of the AP2-like genes, gene24368, due both to its extremely high expression in juvenile seedlings (Figure 3d) and its functional importance in phase transition.

The AP2 family comprises a large class of TFs In plants. The AP2 TFs have been extensively studied in the regulation of cell proliferation, differentiation, adventitious bud development, and integration of age and wound signals [55,56]. Based on these reports and our results, we postulated that AP2-like genes may be involved in the rejuvenation of *R. pseudoacacia* vegetative propagation. Phylogenetic and sequence alignment analysis implied that the AP2-like gene, gene24368, in *R. pseudoacacia* was an ortholog of *TOE1* in *Arabidopsis* (Figures S4 and S5), and we named this gene *RpTOE1*. Subcellular localization suggested that RpTOE1 is a nuclear-localized protein (Figure 5a).

Heterologous overexpression of *RpTOE1* in WT *Arabidopsis* delayed the appearance of abaxial trichomes (Figure S6i,k). Overexpression of *RpTOE1* in *toe1 toe2* double mutants partially rescued the early-flowering phenotype of the mutants and inhibited the expression of *AtFT* (Figure 4c,d,h). The transgenic plants showed a prolonged juvenile phenotype (Figure 4e,f,l). These results suggested that *RpTOE1* does play a role in juvenility maintenance. The quantitative results of *RpTOE1* in different tissues showed that its expression level was the highest in the roots of mature trees and lowest in flowers (Figure S7), which are the organs with high maturity. The fluctuation of *RpTOE1* expression in the aboveground tissues of mature trees can explain to some extent the cyclophysis and topophysis of mature explants during asexual propagation. In juvenile seedlings, the expression level of *RpTOE1* was also the highest in roots, which was consistent with the findings for topophysis, in which basal cuttings were physiologically juvenile [57]. The high expression of *RpTOE1* in roots also confirmed the juvenility of the roots compared to the aboveground parts, and it was feasible to obtain juvenile plants by root asexual propagation.

**Figure 6:**
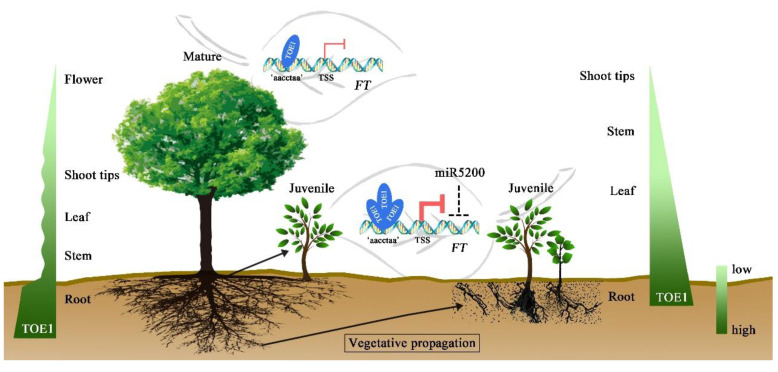
A proposed model illustrating the rejuvenation regulation of *RpTOE1* and its expression patterns in *R. pseudoacacia* root-based vegetative propagation. *RpTOE1* was expressed at a high level in the roots of mature trees and at a low level in the aboveground organs. The expression decreased gradually in the leaves, stems, and shoot tips of juvenile seedlings but fluctuated in these organs in mature trees, and the expression level was lowest in flowers. In the leaves of asexually propagated seedlings, the abundance of *RpTOE1* increased, leading to enhanced inhibition of *RpFT* expression. RpTOE1 binds to the ‘aacctaa’ sequence of the *RpFT* promoter. The thin red arrowed line denotes low-level inhibition, and the thick red arrowed line denotes high-level inhibition. The flowers in the mature trees indicate reproductive ability.

*FT* is not only a regulator of flowering pathways but also an integrator that harmonizes several developmental processes of plants [58]. In *Arabidopsis*, TOE1 represses *FT* expression by directly binding to the promoter and the 3′ downstream regulatory region of *FT* [30,31]. TBS (AACCTACGA), TBS-like (AACCTAAGA, CCTCGAC), and a regulatory element within the *block E* enhancer of *FT* were all potential binding sequences [59]. To further explore how RpTOE1 participated in rejuvenation, we demonstrated that RpTOE1 negatively regulated *RpFT* by directly targeting the TBS-like (AACCTAA) motif of the promoter according to Y1H, EMSA, and dLUC assays (Figure 5b–d). Gene expression and protein-DNA assays suggested that the high expression of TOE1 in *R. pseudoacacia* leaves inhibited the transcription of *RpFT*, resulting in the low expression level of *FT* in clonal plants, which may be the key to maintaining the juvenility of the rejuvenated plants by vegetative propagation (Figure 6). The high expression level of *RpTOE1* in juvenile plants and the low expression level in mature plants can also be used as an indicator of material maturity to a certain extent (Figure 6).

It is worth mentioning that we did not find miR156 and miR172 in the miRNA module (Mblue) that was closely related to phase reversal; these miRNAs are the master regulators of juvenility found in previous studies [21]. We followed the differences between the rejuvenated seedlings and mother trees for two consecutive years in this study, and whether miR156/miR172 are involved in the rejuvenation of root-based vegetative propagation in woody plants needs to be explored with more data in the future. However, miR5200, which was shown to be expressed in leaves and to target *FT* orthologs for mRNA cleavage in *Brachypodium distachyon* [60], was found. Further studies are needed to confirm whether miR5200-targeted *FT* cleavage is involved in the phase reversal of vegetative propagation in *R. pseudoacacia* (Figure 6). Compared with annual plants, woody plants undergo many cycles of environmental changes during their long lifespan and may differ from annual plants in their regulation of rejuvenation. Previous studies showed only that TOE1 inhibits ontogenetic flowering in annual plants, and whether it is involved in phase reversal in woody plants was not revealed. We found that RpTOE1, rather than miR156, plays a central role in the regulation of developmental phase reversal during vegetative propagation in *R. pseudoacacia* mature trees, which was different from the regulation of ontogenetic aging. Furthermore, RpTOE1 interacts with *RpFT* and inhibits its expression to participate in rejuvenation during vegetative propagation in *R. pseudoacacia*. TOE1 was a target of miR172, and miR172 was also positively regulated by the transcription factors that were targeted [61]. Extensive studies need to be conducted to address whether these negative feedback loops contribute to rejuvenation during vegetative propagation and how *TOE1* is highly expressed in rejuvenated seedlings in future studies.

## 4. Materials and Methods

### 4.1. Plant Materials

Three mature wild black locust (*R. pseudoacacia*) trees with different genotypes, located in the Lvcun State-owned Forest Farm of Luoning Country, He’nan Province, China (34°2′40″ N; 111°1′48″ E), were selected as the three clones for vegetative propagation. The ages of the three mature trees (MTs), determined by counting the number of tree rings at breast height (1.3 m), were 21 years old. Two types of root-based asexually propagated seedlings, root sprouting seedlings (RSs) and root cutting seedlings (RCs), were created in early spring 2019. The acquisition of root sprouting seedlings and root cutting seedlings followed the protocols from our previous publication [62]. Sexually propagated seedlings (SSs), of the same age as vegetatively propagated seedlings, were developed from the semi-sib seeds of each mature tree. At least 10 RSs, 10 RCs, and 30 SSs were obtained from each MT. Root cutting seedlings and seed-derived seedlings were grown in a nursery at the same site as root sprouting seedlings. At the same time, in the autumn (7 August) of 2019 and 2020, leaves from the south-facing side of MTs, RSs, RCs, and SSs were collected as one- and two-year-old materials. To reduce the influence of circadian rhythms, all samples were collected from 9:00 to 11:00 a.m. in the morning in both years. For brevity, we used FMT (F means first year), FRS, FRC, and FSS, and SMT (S means second year), SRS, SRC, and SSS to describe the materials collected from MTs, RSs, RCs, and SSs in 2019 and 2020 (Table S4). Leaves collected for RNA extraction were immediately frozen in liquid nitrogen and then stored at −80 °C. At least three independent biological replicates were collected for each sample.

### 4.2. RNA Isolation, Sequencing, and Data Analysis

Total RNA was extracted from leaves using TRIzol reagent (Invitrogen, Carlsbad, CA, USA), according to the manufacturer’s protocol. Sequencing using the Illumina HiSeq X Ten platform (Illumina, San Diego, CA, USA) was performed at IGENECODE, Beijing, China. The sequencing of libraries from each sample with three biological replicates generated an average of 45.92 million paired-end reads per sample. After filtering out adapter and low-quality reads from the raw data, the clean reads were aligned to the genome using HISAT v 2.04 software with default parameters [63]. The transcript expression levels were normalized based on fragments per kilobase of transcript sequence per million base pairs sequenced (FPKM). Differentially expressed genes (DEGs) between mature trees and seedlings within the same year were identified using the DESeq2 R package (1.10.1) [64]. The thresholds for DEGs were set to an adjusted padj < 0.05 and |log_2_(fold change)| ≥ 1.

### 4.3. sRNA Sequencing and Bioinformatic Analysis

The sRNA libraries from each sample with three biological replicates were also sequenced. The raw reads that passed quality control were used in subsequent analyses. The rRNAs, scRNAs, snoRNAs, snRNAs, and tRNAs were removed from the sRNA sequences through a BLASTn search using the NCBI GenBank database (http://www.ncbi.nlm.nih.gov/blast/Blast.cgi/, accessed on 27 March 2022) and the Rfam (11.0) database (http://www.sanger.ac.uk/resources/databases/rfam.html, accessed on 27 March 2022). The filtered sequences were aligned to known plant miRNAs from miRbase 22 (http://www.birbase.org/, accessed on 27 March 2022) using miRDeep2 software [65]. The remaining unannotated reads were used to predict novel miRNAs using Mireap (http://sourceforge.net/projects/mireap/, accessed on 27 March 2022) prediction software by screening the biological characteristics of the miRNAs. The MiRNA expression levels were normalized based on transcripts per million (TPM). Differentially expressed miRNAs (DEMs) between mature trees and seedlings of the same year were analyzed using DESeq2. Thresholds of |log_2_(fold change)| ≥ 1 and false discovery rate (FDR) < 0.05 were considered indicative of significantly different expression. Target prediction of miRNAs was performed with TargetFinder (http://github.con/carringtonlab/TargetFinder, accessed on 27 March 2022) and psRNA Target software (http://plantgrn.nobe.org/psRNATarget/, accessed on 27 March 2022). The default parameters were set for candidate target prediction. The heatmap and expression clusters of differential genes and miRNAs were presented by MeV software after the expression data were normalized by the *Z* score transformation [66].

### 4.4. Weighted Gene Co-Expression Network Construction and Module Detection

We performed WGCNA for gene and miRNA co-expression network construction based on the DEGs and DEMs between the mature trees and at least one type of seedling over two years. WGCNA was based on the hypothesis that genes that have related functions may have similar expression profiles [67,68]. The methods for the construction of these two networks were similar except for the parameters used for co-expression module identification. For the gene network, the parameters for dynamic tree cutting were set to a maxBlockSize of 2000, minModuleSize of 30, and deepSplit of 2. For the miRNA network, the minModuleSize was set to 15 because of the small number of DEMs. The correlations between modules and propagation methods (sexual or asexual) were analyzed. In this study, to distinguish between gene modules and miRNA modules, we referred to gene modules as “G+color” and miRNA modules as “M+color”.

### 4.5. Phylogenetic Analysis

The gene and protein sequences of *Arabidopsis* AP2/ERF were downloaded from PlantTFDB [69]. Multiple sequence alignment of the AP2/ERF TFs and the *R. pseudoacacia* TOE1 protein was constructed by DNAMAN software. A neighbor-joining tree was constructed using MEGA7 by the default method with 1000 bootstraps. The coding sequence of RpTOE1 is available in Dataset S1.

### 4.6. Subcellular Localization Assay

The CDS of *RpTOE1* without a stop codon was amplified by PCR and inserted into the pBI121 vector to generate an *RpTOE1*-GFP fusion construct driven by the 35S promoter (*35S::RpTOE1::GFP*), which was transformed into *Agrobacterium tumefaciens* GV3101. The empty vector was used as the control. The primers for PCR amplification and vector construction are listed in the Table S5. Leaves from 4- to 5-week-old *Nicotiana benthamiana* were used for transient expression by *Agrobacterium*-mediated infiltration. After 2-3 d of culture, florescence signals were captured by confocal laser scanning microscopy (Leica, Weztlar, Germany).

### 4.7. Electrophoretic Mobility Shift Assay (EMSA)

The full-length coding sequence of *RpTOE1* was cloned and inserted into the pET-28a-c (+) vector (Novagen, Darmstadt, Germany) (primer listed in the Table S5). The recombinant RpTOE1 was expressed in *Escherichia coli* Rosetta (DE3), and the fusion protein was purified using PureCube Ni-NTA Agarose (Cube Biotech, Monheim, Germany). Oligonucleotide probes were synthesized and labeled with biotin at the 3′-hydroxyl end (Sangon Biotech, Shanghai, China). An unlabeled probe was also synthesized and used as a competitor. The probe sequence was as follows: 5′- ttggagattcctccattgcaacctaaaatcctttggtggaccaat-3′; the mutated probe (mProbe) was as follows: 5′- ttggagattcctccattgcgggggggaatcctttggtggaccaat-3′. EMSA was performed using a chemiluminescent EMSA kit (Beyotime, Shanghai, China). The reaction in each tube was electrophoresed on a 6% polyacrylamide gel and then transferred to a nylon membrane. The membrane was exposed and imaged under an imaging device (Bio-Rad Gel Doc XR+).

### 4.8. Yeast One-Hybrid (Y1H) Assays

The ~2-kb promoter sequence (designated Pro-FT) of *RpFT* and 300-bp sequence (designated Motif) including the TBS-like motif in Pro-FT were PCR-amplified. The mutated TBS-like motif (mMotif) showed an identical sequence to the TBS-like motif, except that the seven core bases of the TBS-like motif were synthesized. Pro-FT, Motif, and mMotif were inserted into the pAbAi vector (Clontech, Beijing, China) as bait reporters. The CDS of RpTOE1 was cloned into the pGADT7-rec vector (Clontech) as the prey. Yeast one-hybrid (Y1H) assays were performed using the Matchmaker^®^ Gold Yeast One-Hybrid Library Screening System (Clontech, Beijing, China). Bait-reporters were transformed into the Y1hGold yeast strain and selected on Synthetic Dextrose Minimal Medium lacking Ura (SD/-Ura) with 300 ng·ml^−1^ Aureobasidin A (AbA; Sigma-Aldrich, Shanghai, China) to counteract self-activation. Then, pGADT7-*RpTOE1* was transformed into bait-reporter yeast strains and selected on SD/-Leu with or without AbA. pGADT7-p53 + p53-pAbAi were generated in the same manner as the positive control. The primers used are listed in the Table S5.

### 4.9. Dual-Luciferase Reporter Assays

The CDS of RpTOE1 was cloned into the pGreenⅡ 62-SK expression vector as an effector, and the empty vector was used as a control. The *RpFT* promoter (~2-kb upstream) was cloned into the pGreenⅡ 0800-LUC vector to obtain the reporter plasmids. *A. tumefaciens* GV3101 (with pSoup-P19) was transformed with the plasmids for the effectors and reporters. Five- to six-week-old *N. benthamiana* leaves were used as co-infiltrated material. Luciferase activity was measured after 2-3 d of culture using a Dual-Luciferase Reporter Assay System (Promega, E1910). The ratio of firefly LUC to Renilla luciferase (LUC/REN) was used to measure the relative reporter gene expression levels. Six independent replicates were determined for each sample.

### 4.10. RNA Isolation and Reverse-Transcription Quantitative PCR (qRT-PCR)

Total RNA was isolated from frozen samples using the RNAiso Plus Kit (Takara, Dalian, China). First-strand complementary cDNA synthesis for gene quantification was performed with the First-Strand cDNA Synthesis kit (TransGen, Beijing, China) using oligo(dT)_18_ primers. For miRNA quantification, first-strand complementary cDNA synthesis was performed, using miRNA-specific primers. The qRT-PCR and stem-loop qRT-PCR were performed with SYBR green qPCR Master Mix (Tiangen, Beijing, China) for gene and miRNA quantification, respectively [70]. Amplification was performed in a GeneAmp PCR System 9700 (Applied Biosystems, Foster City, CA, USA). All reactions were performed in triplicate. After PCR, the data were computed by the comparative Ct method (2^−∆∆Ct^ method). *Actin* and soybean miR1520d were used as reference genes for gene and miRNA normalization, respectively. The primer sequences are listed in the Table S5.

### 4.11. Generation of Transgenic Arabidopsis

The CDS of RpTOE1 was cloned into the binary vector pROK2 driven by the 35S promoter and introduced into *A. tumefaciens* GV3101. The floral dip method [71] was employed to transfer the *RpTOE1* gene into WT *A. thaliana* (Col-0) and *toe1 toe2* double mutants. Seeds were supplied by the Nottingham Arabidopsis Seed Centre (NASC). All seeds were harvested and screened for positive plants on MS medium containing kanamycin (50 mg·L^−1^). The positive plants were verified by genomic PCR and qRT-PCR quantification of the *RpTOE1* gene. T_3_ transgenic plants with a single transgenic copy were used for subsequent experiments. All of the *Arabidopsis* plants were grown in an artificial climate chamber under long-day conditions (16 h light/8 h dark, 22 ℃). The flowering-related traits were measured in three independent homozygous transformed lines, and at least 30 events were counted. The significance of differences between transgenic and mutant or WT plants was analyzed by Student’s *t*-test. The primers for plasmid construction, PCR, and qRT-PCR are listed in the Table S5.

### 4.12. Availability of Data and Materials

Any reasonable requests are available from the corresponding author.

### 4.13. Accession Numbers

The accession numbers of *Arabidopsis* lines mentioned in this article are as follows: *AtFT* (At1g65480); Salk_069677 (*toe1–2*); and Salk_065370 (*toe2–1*).

## Data Availability

The datasets generated and/or analyzed during the current study have been deposited in the NCBI Sequence Read Archive repository under accession numbers PRJNA742908, PRJNA765931, PRJNA765511, and PRJNA765528 (https://www.ncbi.nlm.nih.gov/bioproject, accessed on 27 March 2022).

## Supplementary Materials

The following supporting information can be downloaded at: https://www.mdpi.com/article/10.3390/ijms23095079/s1.
