# Supplementary material for: The RpTOE1-RpFT Module Is Involved in Rejuvenation during Root-Based Vegetative Propagation in Robinia pseudoacacia
Source: Int J Mol Sci. 2022 May 3;23(9):5079. doi: 10.3390/ijms23095079 (PMC9104387; doi:10.3390/ijms23095079)
Supplement: Supplementary file 1 [file ijms-23-05079-s001.zip › ijms-1678312-Supplemental Dataset S1-done.pdf]

>*Robinia pesudoacacia* gene24368 CDS (*RpTOE1*, GenBank accession number: 2511839)

atgttagatcttaatctgaatgccgattcgactcagaacgatgactcgctcgtcgtgttgga-
taagtttccagaagcatcttctggaacctccaattcctccatcgtgaatgctgaggggtcaagtaacgaggactcgtgttccacac
gcgccggggacgcgttcacctacaattttggtatccttaaggtggaaggagggaac-
ggcgtcgttgcaaccaaggagcttttcccggtgcagccttcaacgtcttcgattttggcaaggaagagcttggtggatctctcgct
ggatcatcatcatcgaggccaaaacgacgacgttaatttggttcaggttcag-
caacaacagcctcaggcgaagaagagtaggagaggtccaaggtctcggagttctcagtatagaggagtcaccttctatagaag
aactggaagatgggaatcgcatatctgggattgcgggaaacaagtctatttgggtggattt-
gacactgctcatgccgctgctagagcctatgatcgagctgctatcaagttcaggggacttgatgctgacatcaatttcaatctcgtt
gattatgaggaggatatgaaacagatgacaaatctttccaaggaggaattcgtg-
cacatactacgtcgccacagtaccggtttctcaaggggggagctcaaaataccgaggagtgacacttcacaaatgtggccgttgg
gaagctcgaatgggacaattccttggcaaaaaggcttatgacaaggcagctatcaagtg-
caatggaagagaggcagtgactaacttcgagccaagtacatatgaaagcgagatgaaaccggaagctattaatgaaggtagc
agtcacaatcttgacctcaatttgggcatagcaaccccaggacatggtccaaaa-
gaaaacagggggcatcttcagttccagtccgtcccttacaacttgcaccctggaagaagttcaaggatggagactaatgttaatt
cagttatcggtgatccatctttgaaaaggctcgttgtaactgaagagcgtccttctg-
tatggaatgccacgtattccagtttctttcccagtgaggaaagagcagagagaatgggcatagatccttcggaaggactccgca
actgggcgtggcaaacacatggccaggtcactgctaccccagttccaccgttctctagtg-
cagcatcatcaggattctcaatttcagctacctttccatccactgccatctttccaacaaaatctacgaactcaattccccagagcatc
tgtttcacttcatccagtgcatctggtaacaatgcagctcaatacttctaccaggtgaagtccccgcaagcaccaccctag

>Robinia pesudoacacia FT promoter sequence

gaatctaataattagcaacttattcctcgtgggttcgataattcgg-
gacttaaaatcctttattactattgcgatagagtgcacttgctcttagtgtgttttatgcgtaacaaaagttcagttgcaaagccacca
aaaattataaaaataccttgcaactaggttgaagtatgcaagggtcgatcccatgag-
gaactcaggtaattatcaatgtgaagtgttcttgttcctaactcttttaaataacaaaaactgtaaaggtacagggagtttaagtg
aatgcttgactaaaatctaaaagaacaattaatcaaaacaataagtaaagtt-
gatgaaaacttcttatggaaagacttggttagggaatataaatccatactagattgcatttttattcattcatttccgattgcttgactg
gccaaatggacaccaaaagagctgcaatgatgttcactgatataacgggtggtgaggag-
tatgttattggcagacccagagggtccattccaggcagtgccacagtggaggttggagattcctccattgc**<u>aacctaa</u>**aatccttt
ggtggaccaatttggaactaatgacgactcttcaagtcta-
gatggctctgaacccacccccaaaaacagaggatagatgaccgagggaagtccatggtggtggtcaattttgaggaagagac
tccgttggaactacaatctcgtcccccacagtccctgtcaatcctctctaatcag-
gatcacacatctctgcgaggagcttctactgatgagctcaaagtcatgttttggcagagtgctcatgtttcgctttttgtcatgaca
atattgacaagattgccagatttcctgccatagaaggcgagctggag-
gaagttaaggctgaaaaagcaaagcttgaggatcaaaagaagattttggacgacaagccgaaatcttctgatcaaacaatcct
gtagttgcgcgagaagatctggcttcttcaattggaggttgaagctgagaagaaacaaaaa-
gaaaaggcctttactgaacttgagggtgagaaatctgctaagttggaagttgaggcgactgttgaatattggatgggtgatgtg
aagaccactaacggcttcaacaatgctctaaggcagacagagtttttcaacaaggactt-
gtcaatcaatcttgagcacttccatccttggcgctttattgatgacaaggggcagttaaatgaatatcggtagggcatcgcccatgt
catcgag-
tggcccctgacatccgattgagcctgtttttttatgtattttttaaaaaaatttgttttgtttttttgttttgtgatatttttttgtttttttttt
attcataatgaattatattttgtatttgcttattcataatgaattctgtttttcac-
gtactgaaaatttttaaactgttttcaatccagtagcacgaaaaacgaattctaattgtaacaatccaattcggttttatctccacctg
aatctaaaataaaacatattgcgatttagtaggggaaaacgaattgattgctatgtaacat-
acgatatggttacatataaccgaatcataatgttgaaacatgcaatttggttatttgtaaccgtatcgtatgttacatagcaatcaatt
cattttcccctaccgaattgaattcaacatatcattcaattcagtatctcgttaaac-
gaattttatgcacactcaaagggattagggaattaatataagaaatataattaaatttaaattactagctttttattatttaaagttgta
ttaaattctaccagggaattaatacgttcctaattacgtactttatatacacttaaa-
gaaaaaaagattgctagacaacacaggcaaggaatatccagacccctcctcccccctctataaatagcttgcctttggtggttatag
tagcactcgtgtgcccaaagccaaatttgcatatagcagtgtgagccagtgagattgaatc

Note: The TBS-like motif was in bold and underlined.

>TBS-like motif sequence for Y1H

atttttattcattcatttccgattgcttgactggccaaatggacaccaaaagagctg-
caatgatgttcactgatataacgggtggtgaggagtatgttattggcagacccagagggtccattccaggcagtgccacagtgg
aggttggagattcctccattgc**aacctaa**aatcctttggtggaccaatttggaactaatgac-
gactcttcaagtctagatggctctgaacccacccccaaaaacagaggatagatgaccgagggaagtccatggtggtggtcaatt
ttgaggaagagac

Note: The TBS-like motif was in bold and underlined.

>TBS-like mMotif sequence for Y1H

atttttattcattcatttccgattgcttgactggccaaatggacaccaaaagagctg-
caatgatgttcactgatataacgggtggtgaggagtatgttattggcagacccagagggtccattccaggcagtgccacagtgg
aggttggagattcctccattgc**ggggggg**aatcctttggtggaccaatttggaactaatgac-
gactcttcaagtctagatggctctgaacccacccccaaaaacagaggatagatgaccgagggaagtccatggtggtggtcaatt
ttgaggaagagac

Note: The mutated TBS-like motif was in bold and underlined.
