# Supplementary material for: The RpTOE1-RpFT Module Is Involved in Rejuvenation during Root-Based Vegetative Propagation in Robinia pseudoacacia
Source: Int J Mol Sci. 2022 May 3;23(9):5079. doi: 10.3390/ijms23095079 (PMC9104387; doi:10.3390/ijms23095079)
Supplement: Supplementary file 1 [file ijms-23-05079-s001.zip › ijms-1678312-Supplementary Figures-done.pdf]

International Journal of
Molecular Sciences

MDPI

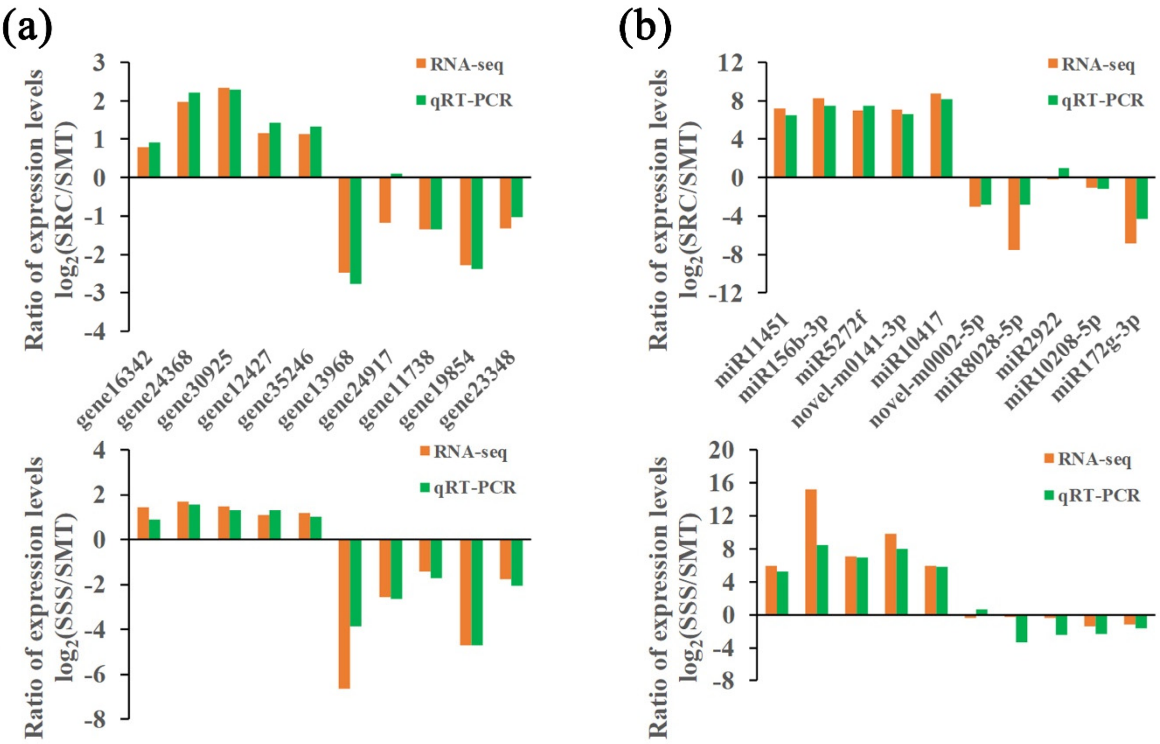

Figure S1. Expression ratios of selected DEGs and DEMs as determined by qRT-PCR and deep sequencing. (**a**) DEGs determined by qRT-PCR and deep sequencing; (**b**) DEMs determined by qRT-PCR and deep sequencing.

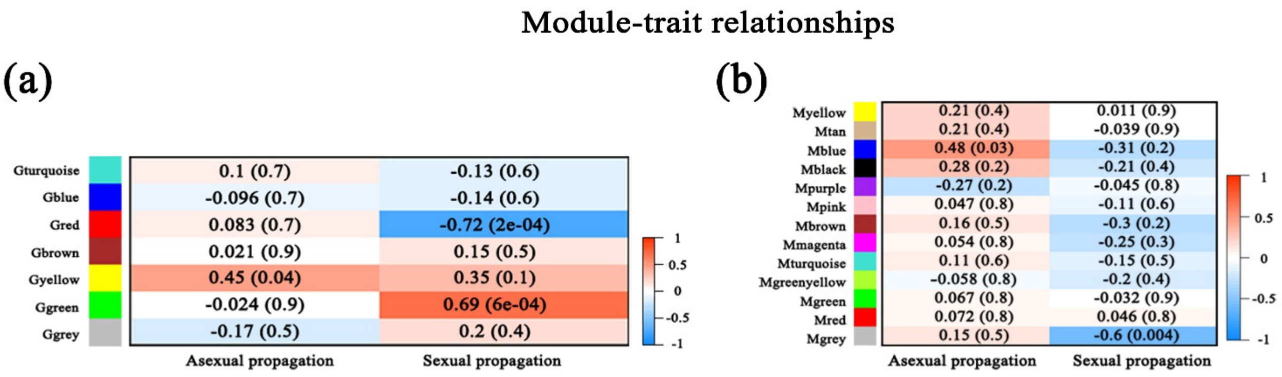

Figure S2. Correlations between modules and traits in WGCNA. (**a**) Correlations between gene modules and the sexual or asexual propagation of seedlings in WGCNA; (**b**) Correlations between miRNA modules and the sexual or asexual propagation of seedlings in WGCNA. The numbers in the module represent correlation coefficients and the numbers in parentheses represent p values.

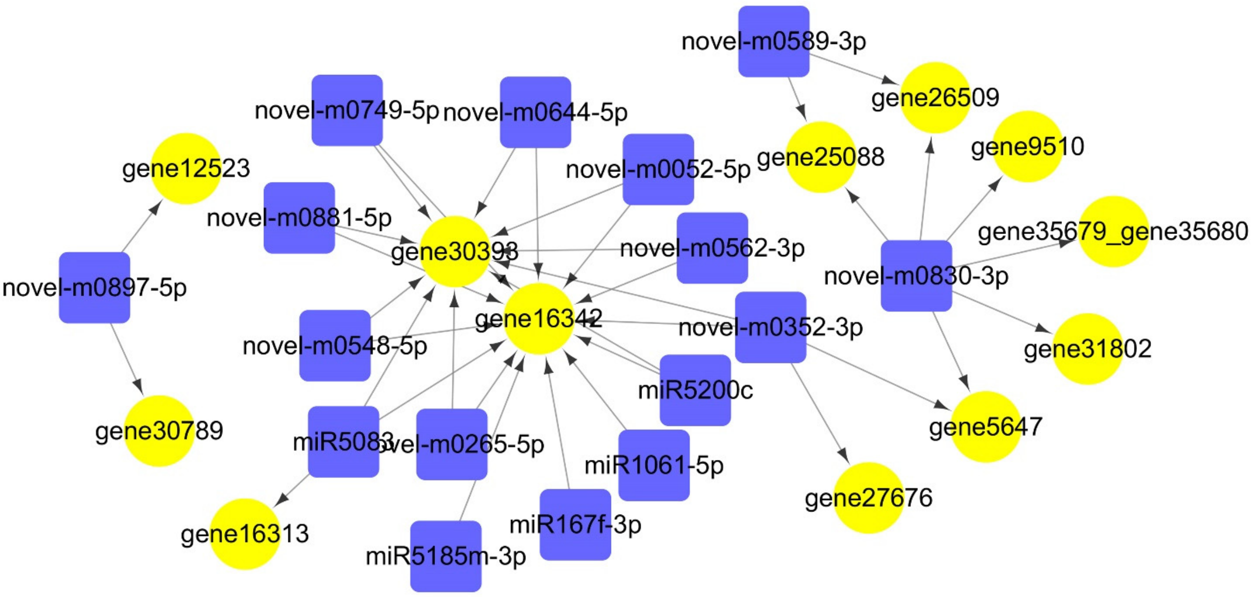

Figure S3. Target prediction between genes in Gyellow and miRNAs in Mblue.

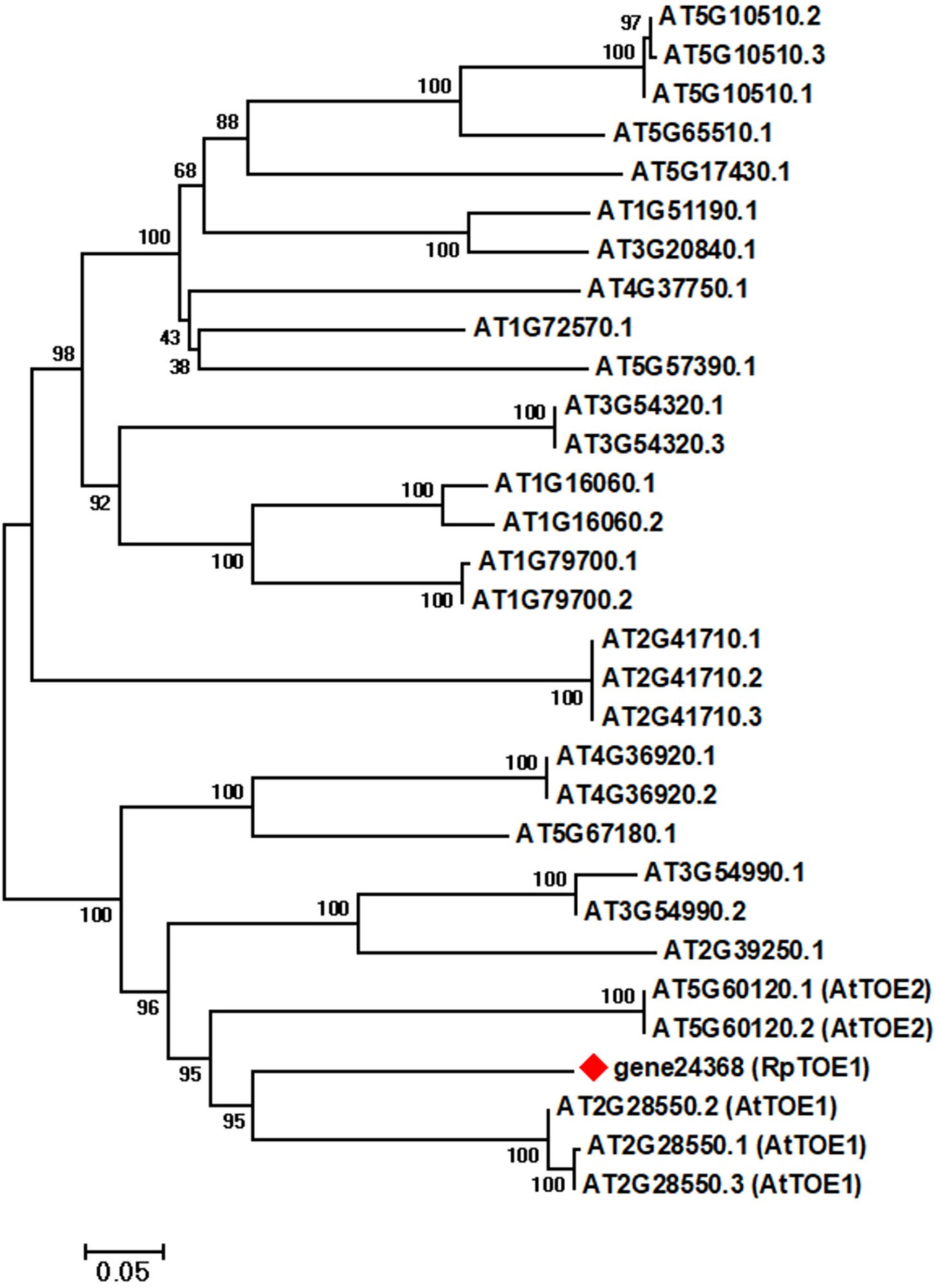

Figure S4. Molecular phylogenetic analysis of gene24368 in the *Arabidopsis* AP2 subfamily using the neighbor-joining method with 1000 bootstrap replicates in MEGA7.

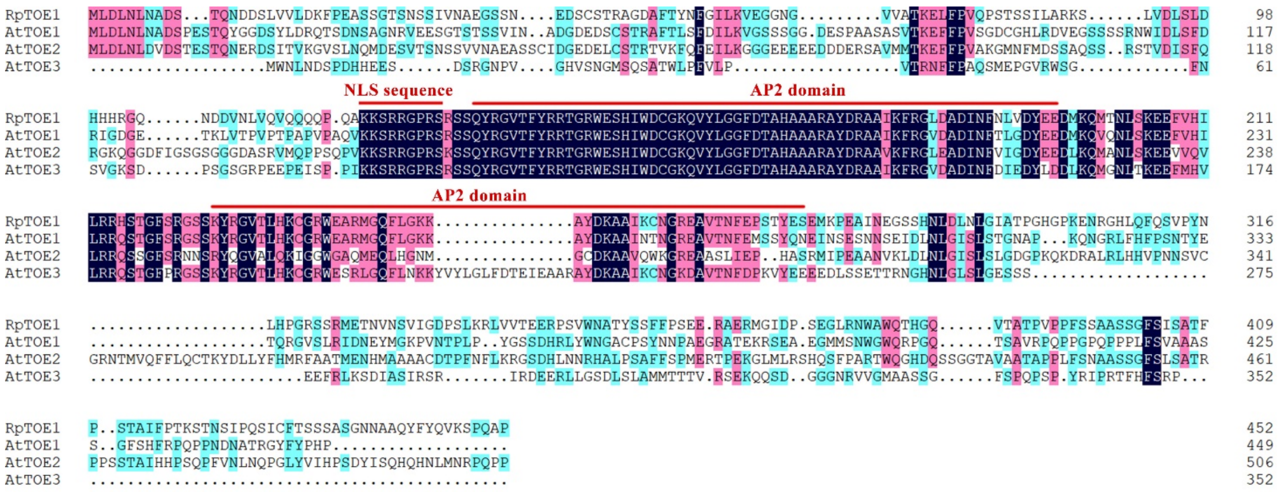

Figure S5. Analysis of the conserved domains of RpTOE1.

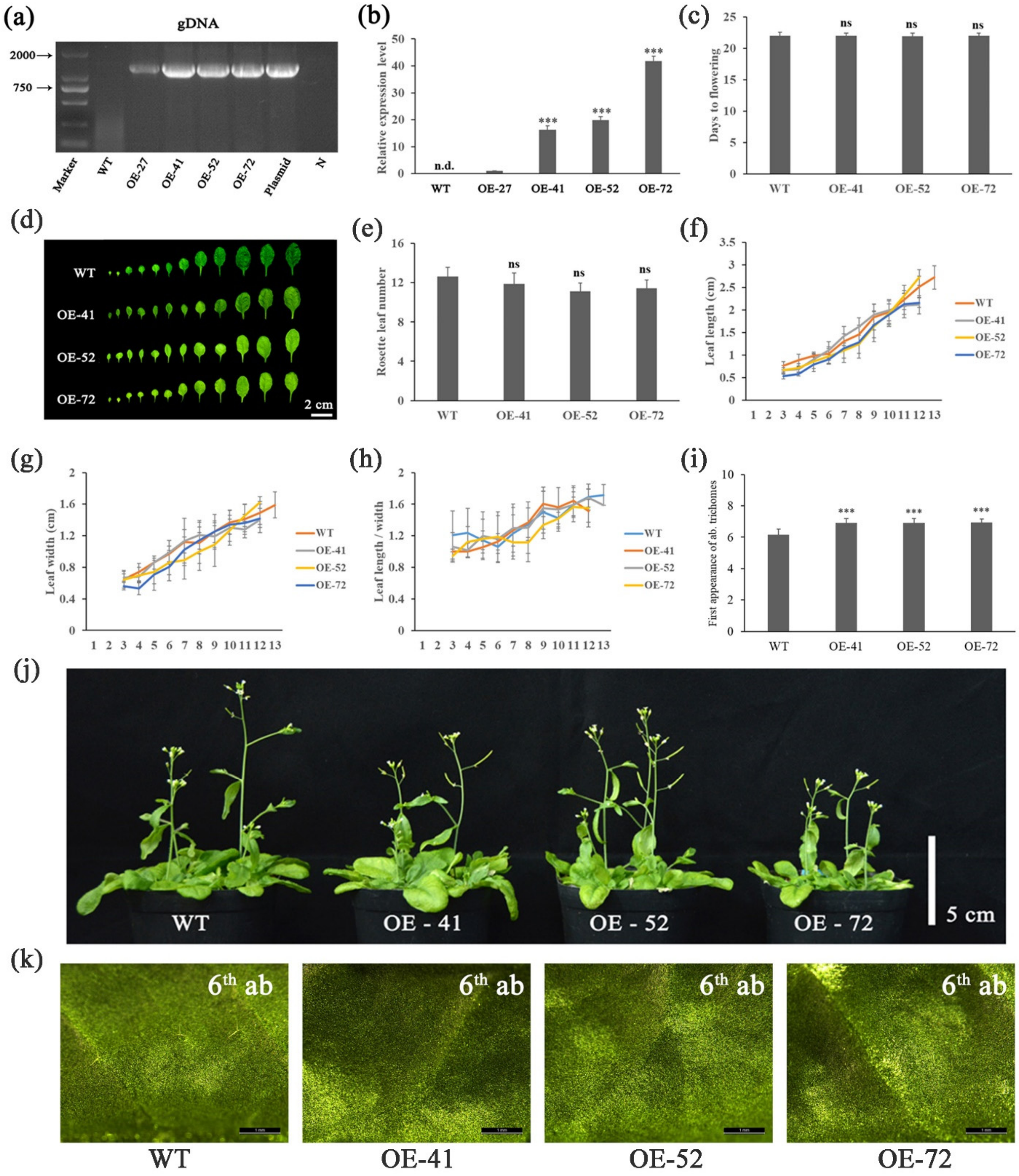

Figure S6. Overexpression and identification of *RpTOE1* in wile-type *Arabidopsis* (WT). (**a**) PCR analysis of *RpTOE1* genes using genomic DNA from WT and four transgenic lines; (**b**) Relative expression of *RpTOE1* in WT and four transgenic lines with *AtACTB* as an internal control using qRT-PCR. Data represent the average of three biological replicates; (**c**) Days to flowering of WT and transgenic lines; (**d**) All rosette leaves on one plant of WT and transgenic lines when floral primordia were visible in WT; (**e**) Rosette numbers when floral primordia were visible; (**f–h**) Leaf shape measurement when floral primordia were visible. The x-axes indicate the order of leaves. The y-axes indicate leaf length (**f**); leaf width (**g**); and leaf length/width ratio (**h**); (**i**) The abaxial (ab) trichome statistics. The first leaf with abaxial trichomes was scored ($n > 20$); **j** Phenotype of the *RpTOE1*-overexpressing plants and WT controls; (**k**) The abaxial trichome phenotype. N, negative control using water as a template. n.d., not detected. ns, not significant. The expression levels were quantified in 15-day-old seedlings. *** significant differences between the OE-27 line based on Student's *t*-test ($p < 0.001$). n.d.

= not detected. ns = not significant. Error bars represent SEM. Bar = 2 cm in (**d**), bar = 5 cm in (**j**), and bars = 1 mm in (**k**) $n > 30$ in (**c**, **e**, **f**, **g**) and (**h**).

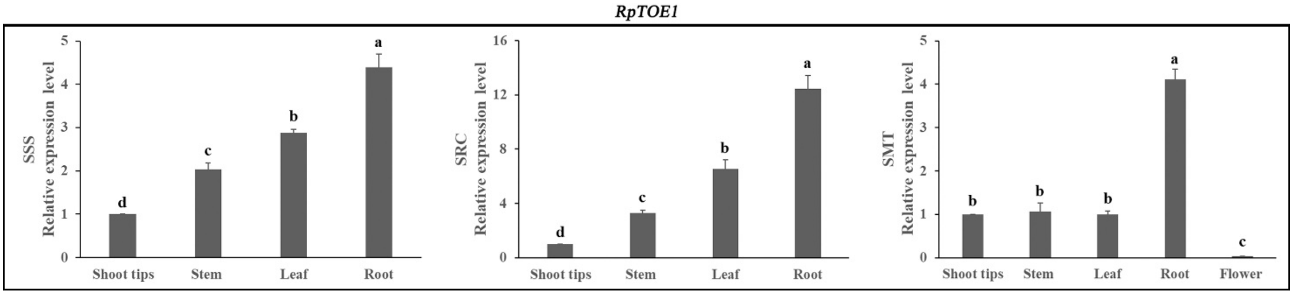

Figure S7. *RpTOE1* levels in different plant tissues. SSS, two-year-old seed-derived seedlings. SRC, two-year-old root cutting seedlings. SMT, mature trees in the second year of sampling. The expression average is from three independent biological replicates. Bars are the mean ± SD. Lowercase letters designate groups that significantly differ ($p < 0.05$).
